# Predicting the level of job satisfaction based on hardiness and its components among nurses with tension headache

**Published:** 2015

**Authors:** A Mahdavi, E Nikmanesh, M AghaeI, F Kamran, Z Zahra Tavakoli, F Khaki Seddigh

**Affiliations:** * Department of Psychology, Faculty of Psychology and Educational Sciences, University of Payame Noor, Tehran, Iran; ** Department of Psychology, Family Therapy, University of Shahid Beheshti, Iran; *** Department of Psychology, Faculty of Psychology and Educational Sciences, University of Tehran, Tehran, Iran; **** Department of Psychology, Islamic Azad University, Saveh Branch, Iran; ***** Department of Psychology, Faculty of Psychology and Educational Sciences, Islamic Azad University, Karaj Branch, Iran

**Keywords:** job satisfaction, hardiness, tension headache, nurses

## Abstract

Nurses are the most significant part of human resources in a sanitary and health system. Job satisfaction results in the enhancement of organizational productivity, employee commitment to the organization and ensuring his/ her physical and mental health. The present research was conducted with the aim of predicting the level of job satisfaction based on hardiness and its components among the nurses with tension headache. The research method was correlational. The population consisted of all the nurses with tension headache who referred to the relevant specialists in Tehran. The sample size consisted of 50 individuals who were chosen by using the convenience sampling method and were measured and investigated by using the research tools of “Job Satisfaction Test” of Davis, Lofkvist and Weiss and “Personal Views Survey” of Kobasa. The data analysis was carried out by using the Pearson Correlation Coefficient and the Regression Analysis. The research findings demonstrated that the correlation coefficient obtained for “hardiness”, “job satisfaction” was 0.506, and this coefficient was significant at the 0.01 level. Moreover, it was specified that the sense of commitment and challenge were stronger predictors for job satisfaction of nurses with tension headache among the components of hardiness, and, about 16% of the variance of “job satisfaction” could be explained by the two components (sense of commitment and challenge).

## Introduction

Tension type headaches (TTH) are returnee episodes of headache persistent for hours to weeks. The throe is typically tightening or pressing in quality, of minor to average intensity, and reciprocal in situation, and does not deteriorate with the routine physical activity. They do not have the nausea and vomiting. Nevertheless, they may have phono‌phobia and photophobia (ICHD, 2004). The tension-type headache is the most commonplace and expensive headache [**1**]. Headaches are classified into two categories: this disorder, previously called muscle contraction headache or tension headache, is a common ailment generally self-treated with over-the-counter (OTC) analgesics; commonplace rates of tension-type headaches are diverse among studies from 291 to 712 percent of the clients examined, due to differences in research study design [**2**,**3**] .

In the last 30 years, there has been a diverse research interest in the concept of “hardiness” [**4**], which suggested that hardiness is a healthy personality disposition including three elements: control, challenge, and commitment. A though individual is the one who views events that could be potentially stressful as alluring and significant (i.e., commitment), observe oneself as capable of altering circumstances (i.e., control), and perceive alter as normal and as an opportunity for improvement (i.e., challenge). Ample research evidence has supported Kobasa’s reasonableness that hardiness is a healthy personality disposition [**2**,**5**]. Job satisfaction is a vital challenge for the healthcare organizations, especially among nurses. Since the financial resources are always limited and demand a high level of expenses, there has been an increasing demand for nurses but there is not enough supply to meet this demand [**6**]. Shortage of nurses and quitting this profession is going to be a global challenge that is important in both the developing countries and the developed ones. The related studies demonstrated that the lack of job satisfaction is an important factor in quitting the job. Moreover, one third of the nurses in England and Scotland and one fifth of the nurses in the USA tend to leave the job [**7**]. According to Monjamed Z, Ghorbani T, Mostofian, Oveysipoor R, Nakhost Pandi S, Mahmody M [**8**], long working hours, work conditions, poor evaluation practices, and shortcomings in the way of rewarding and punishing are the cause of this dissatisfaction [**8**].

Locke EA [**9**] explained the concept of job satisfaction as an enjoyable position of a positive emotional situation resulting from job experiences or the evaluation of one’s work. Job satisfaction is the result of a personal appraisal of job and career experiences. Van der Ploeg JD, Scholte EM [**10**] differentiated five fundamental elements of job satisfaction: autonomy, management support, essence of work, relations with colleagues, and working conditions. They used the job satisfaction index (ASI) to congregate information on each of these elements. When job satisfaction decreased, phenomena such as work-related stress and fatigue could become apparent. While work-related stress is generally impermanent, burnout refers to a more persistent tribulation. Fatigue is a condition in which individuals are unloaded of mental and physical vigor due to an exaggerated endeavor to perceive appropriate expectations either set by themselves or by their superiors, or due to a desire to act in agreement with the social norms at a time when the needs and wants of the self are not being met [**11**]. Healthcare organizations have a special position in societies due to their important task in the field of prevention, care, and treatment. Moreover, job dissatisfaction of the employees in healthcare centers would lead to the low quality of the provided services and consequently to the dissatisfaction of the patients. Hence, according to the extent of the nursing department in the health and education centers in the country, paying a special attention to their opinions and demands is indeed paying attention to the health of the society. Since the increase of the nurses’ job satisfaction in hospitals resulted in the enhancement and promotion of the healthcare services, eventually, it led to the improvement of the patients’ satisfaction regarding the healthcare services. Moreover, considering the key role of nurses in presenting healthcare services to the patients was of the important factors in the improvement of the presented service. Therefore, conducting scientific studies in this area and using the results in various aspects of nursing could lead to useful consequences. According to the mentioned issues, the present research was conducted with the aim of predicting the level of job satisfaction based on hardiness and its components among the nurses with tension headache who referred to the hospitals in Tehran in 2015.

## Methodology

The present research method was correlational. The population consisted of all the nurses with tension headache who referred to the relevant specialists in Tehran in 2015 in order to receive pharmacotherapy and psychotherapy services. The sample size consisted of 50 individuals who were chosen by using the convenience sampling method and were measured and investigated by using the research tools of “Job Satisfaction Test” of Davis, Lofkvist and Weiss and “Personal Views Survey” of Locke EA [**9**].

**Personal views survey**

The personal views survey of Locke EA [**9**] was used in order to measure hardiness. This survey consisted of 50 items including the subtests of challenge (17 questions), commitment (16 questions) and control (17 questions), which were formed based on a 4-point Likert scale (from false to completely true). Maddi SR, Kohn S, Maddi KL [**12**] analyzed the factors of this survey and found that there was an acceptable reliability and validity for “hardiness”. Other related researches indicated that each of the components of commitment, control and challenge had the least reliability while most of the reliability coefficients, whether for the subordinate scores or the whole characteristic of hardiness, were more than 0.8 [**13**].

**Job Satisfaction Test of Davis, Lofkvist and Weiss**

This test was developed by Davis, Lofkvist and Weiss in 1977 and consisted of 20 questions based on a five-point Likert scale (very satisfied, satisfied, I don’t know, dissatisfied, very dissatisfied). Various researches demonstrated a high-level reliability and validity for the test. Rezaie (1998) calculated a 0.83 value for the reliability coefficient. In addition, the reliability coefficient obtained was equal to 0.87, 0.86, and 0.85 in the studies conducted by Hemati Rad G [**14**] and Shafiabadi A [**15**], respectively. The statistical method of the Correlation Coefficient was used to analyze the obtained data. Moreover, the Regression Analysis method was employed in order to predict job satisfaction from the various components of hardiness.

**Findings**

**Table 1:** Pearson correlation coefficient for the variables of hardiness (its components) with job satisfaction among the nurses

Variables	Job satisfaction	Hardiness	Commitment	Challenge	Control
Job satisfaction	1	**0.506	**0.482	*0.276	*0.479
Sig. Level		0.000	0.000	0.025	0.000
Hardiness	**0.506	1	**0.871	**0.758	**0.780
Sig. Level	0.000		0.000	0.000	0.000
Commitment	**0.482	**0.871	1	**0.472	**0.657
Sig. Level	0.000	0.000		0.000	0.000
Challenge	**0.279	**0.785	**0.472	1	*0.297
Sig. Level	0.025	0.000	0.000		0.000
Control	**0.482	**0.780	**0.657	*0.297	1
Sig. Level	0.025	0.000	0.000	0.016	
** Correlation is significant at 0.01 level			** Correlation is significant at 0.05 level		

The findings in the **table** above indicated that the calculated correlation coefficient for hardiness and job satisfaction was 0.506 and this coefficient was significant at the 0.01 level. In other words, it could be expressed with a 99% confidence level that there was a significant and positive relationship between the hardiness and job satisfaction of nurses. Moreover, there was a correlation between the job satisfaction and each of the components of hardiness (commitment (0.482), challenge (0.279), and control (0.479)). The correlation coefficients obtained for job satisfaction and commitment (0.482) and job satisfaction and control were significant at the 0.01 level, and the obtained coefficient for the relationship between job satisfaction and challenge was significant at a 0.05 level. Therefore, it could be expressed that there was a positive and direct relationship between job satisfaction and each of the components of hardiness (commitment, challenge, and control) among the nurses.

**Table 2:** Regression of job satisfaction based on the dimensions of psychological hardiness

Dependent variable	β	t	P
Commitment	0.31	04.01	0.02
Challenge	0.27	4.29	0.01
Control	0.12	0.62	NS
R		0.39	
R2		0.17	
Adjusted R2		0.16	
Criterion error		19.75	
F		19.02	

The multivariate regression method was used to study the prediction ability of the dimensions of psychological hardiness in predicting job satisfaction. Based on the findings in **Table 2**, the sense of commitment had the most importance in terms of predicting the job satisfaction with the beta value equal to 0.31 and the challenge located in the next level with the beta value equal to 0.27. According to the above table, 16% of the variance of job satisfaction could be predicted through a sense of commitment and challenge.

## Discussion and Conclusion

The research findings demonstrated that there was a significant and positive relationship between the psychological hardiness and each of its components (means commitment, challenge, and control) with job satisfaction. In other words, the higher levels of hardiness led to greater job satisfaction. The relationship between the components of hardiness (commitment, challenge and control) and job satisfaction among the nurses indicated that there was a positive and significant relationship between job satisfaction and each of the components of hardiness (commitment (0.482), challenge (0.279) and control (0.479)). The relationships of commitment and control with job satisfaction were significant at a 0.01 level and the relationship between challenge and job satisfaction was significant at a 0.05 level. However, the role of commitment was more than the components of control and challenge. Commitment referred to being important, interesting, and valuable and the significance of the activities of life. It seemed that having belief in being important, being interesting and significant in the professional activities, had a greater impact on job satisfaction than the belief in controlling the atmosphere and preparedness for dealing with career changes.

Psychological hardiness led to the enhancement of hope in individuals through providing resistance against stress and appropriate coping strategies, which expressed more adaptability of hopeful individuals with stress and the ability to moderate stressful events. Hard individuals usually have a better state of well-being due to the use of active and effective coping strategies. These people deem unpleasant conditions challenging rather than threatening, feel a greater sense of commitment to their work, experience a greater sense of control in their life and consider stressful factors as potential opportunities; therefore, they can maintain their mental health and adjustment. Nurses who have a greater sense of commitment continue their job with a great interest, deem it as an important and interesting situation in their life, and do their duties significantly. Moreover, the findings demonstrated that job satisfaction could be predicted by using hardiness and its dimensions. In other words, psychological hardiness provides the possibility of predicting the individuals’ job satisfaction because hard people consider their work stresses less stressful. Therefore, they can have a more effective adjustment to their job. Among the components of hardiness, challenge had the most importance. Challenge is the interface between hardiness and job satisfaction because individuals with higher levels of satisfaction are more flexible. The present finding was somehow the most important finding of the present research and was indicated as the most important of hardiness, as a positive characteristic in job satisfaction of nurses and the enhancement of their adjustment. In addition, the finding was significant for the consultants because it showed that they could help nurses to increase job satisfaction and to resolve conflicts by emphasizing hardiness. In order to infer this conclusion, it could be expressed that several factors were effective in increasing job satisfaction and one of these factors was personality characteristics. Psychological hardiness is a personality characteristic. Using the conceptual and theoretical relationship, existence of the significant relationship between hardiness and job satisfaction could be perceived and explained. It could be expressed that psychological hardiness is a personality construct, which makes the individuals not only not consider the present situation threatening and uncontrollable but also make them perform activities they are not interested in. Using some strategies, they could change the activities to an interesting and positive work and an opportunity to growth and development. Therefore, they could change the situations in accordance to their characteristics.
